# Characterizing the symptomatology and pathophysiology of allergic rhinitis using a nasal allergen challenge model – a subset of the allergic rhinitis microbiome study

**DOI:** 10.1186/s13223-025-00980-5

**Published:** 2025-08-18

**Authors:** Sophia Linton, Lubnaa Hossenbaccus, Abigail Davis, Jen Thiele, Sarah Garvey, Hannah Botting, Lisa Steacy, Anne K. Ellis

**Affiliations:** 1 Division of Allergy & Immunology, Department of Medicine, Queen’s University, Kingston, Canada; 2 Allergy Research Unit, Watkins 1D, Kingston Health Science Centre – Kingston General Hospital Site, 76 Stuart Street, Kingston, ON K7L 2V7 Canada; 3 Department of Biomedical and Molecular Sciences, Queen’s University, Kingston, Canada

**Keywords:** Allergic rhinitis, Nasal allergen challenge, Eosinophils, Immunoglobulin-E

## Abstract

**Background:**

Since 2015, our nasal allergen challenge (NAC) protocol has been used to investigate the pathophysiology of allergic rhinitis (AR) with various allergens. However, we have yet to publish a comprehensive examination of the pathophysiology associated with AR to ragweed pollen.

**Methods:**

Nineteen ragweed pollen allergic and 12 healthy (nonallergic) control participants from Kingston, Ontario, Canada, completed the NAC to ragweed pollen extract out-of-season. Total nasal symptom score (TNSS) and percent fall in peak nasal inspiratory flow (PNIF) were collected up to 48 h post-exposure. Nasal fluid and serum samples were collected post-exposure, and white blood cell differential counts, serum ragweed-specific and total immunoglobulin-E (IgE), and nasal cytokine concentrations were analyzed. Statistical tests were performed using GraphPad Prism 10.4.0.

**Results:**

The mean TNSS and percent PNIF fall from baseline were significantly higher in participants with ragweed pollen allergy compared to nonallergic controls up to 24 h (*P* ≤ 0.05) and 12 h (*P* ≤ 0.05) post-NAC, respectively. Nasal eosinophils significantly increased in allergic participants at 6 h (*P* = 0.0010) and 24 h (*P* = 0.0049), while peripheral blood eosinophil percentages decreased significantly at 6 h compared to baseline (*P* = 0.0499). The specific to total IgE ratio for allergic participants significantly increased 1 h and 24 h (*P* = 0.0022 and *P* = 0.0034, respectively) post-NAC, with a decrease at 6 h compared to both 1 h and 24 h (*P* = 0.0224 and *P* = 0.0316, respectively). Allergic and nonallergic participants had significantly different cytokine profiles, particularly IL-4, IL-5, IL-6, IL-13, MIP-1β, and TNF-α.

**Conclusions:**

This study confirms the effectiveness of our NAC protocol in eliciting clinical and biological responses in ragweed-allergic participants, particularly highlighting eosinophil activity, IgE, and cytokine dynamics. Future research should investigate the roles of specific IgE, IL-4, and eosinophil activation in allergic inflammation. Additionally, this NAC study population provides a strong foundation for examining the nasal microbiome in AR. Longitudinal studies exploring the relationship between allergic responses and microbiome shifts could offer deeper insights into the underlying mechanisms of disease.

**Supplementary Information:**

The online version contains supplementary material available at 10.1186/s13223-025-00980-5.

## Background

The prevalence of allergic rhinitis (AR) varies globally, affecting between 5% and 50% of the population [1]. In Canada, it is estimated that up to 20% of individuals are diagnosed with AR [2]. This condition is characterized as an immunoglobulin E (IgE)-mediated, type 1 hypersensitivity reaction of the nasal mucosa triggered by allergen exposure in sensitized individuals [3]. Upon exposure to allergens, sensitized patients produce allergen-specific IgE (sIgE), initiating both early- and late-phase reactions during subsequent exposures [4, 5]. This ultimately leads to the clinical symptoms of AR, which include rhinorrhea, nasal congestion, nasal pruritus, and sneezing [3]. While various treatment options are available, including pharmacotherapy and allergen immunotherapy, up to 60% of AR patients are dissatisfied with their treatment [2]. This emphasizes the need to explore and comprehend the pathophysiology of AR further to aid in the development of novel and more effective treatments.

Experimental disease models can be utilized to investigate the pathophysiology of AR, one of which is the nasal allergen challenge (NAC). This procedure involves administering an aerosolized dose of allergen into the nasal cavity through both nostrils and monitoring the response. Numerous NAC studies have shaped our current understanding of the pathophysiology of AR. Within minutes of allergen provocation, increases in several mediators are observed locally and systemically, including histamine, tryptase, prostaglandins, and leukotrienes, typically released from mast cells [6–11]. In patients with AR, pronounced nasal symptoms following a NAC can be detected within 15 min [12–14]. The response to a NAC may persist for up to 24 h, with symptomatic patterns varying significantly among individuals [12]. Notably, increases in the levels of type 2 inflammatory cytokines can be seen in nasal secretions within minutes post-NAC, lasting up to 24 h [6, 15–22]. IL-5, in particular, has been associated with eosinophils in the nasal mucosa and correlates with nasal symptoms following the NAC [6, 23]. There is also a notable influx of inflammatory cells into the mucosa after a NAC, including eosinophils [12, 23–25], basophils [26], T lymphocytes [18, 27, 28], and various antigen-presenting cells (APC) such as monocytes, dendritic cells, and group 2 innate lymphoid cells (ILC2s) [29–32]. A systemic allergic response may develop after the NAC, with elevated levels of serum sIgE against the allergen detectable within a week, and these levels may remain elevated for up to a month [12, 33]. Additionally, following allergen exposure, we see increased activation of circulating basophils, along with a rise in the counts of blood eosinophils, neutrophils, lymphocytes, and ILC2s within hours [34–37]. Levels of serum cytokines, including IL-5 and IL-13, are also upregulated after a NAC [38]. 

Blackley first introduced the NAC in the late nineteenth century, and it has been used prolifically in AR research since then [39, 40]. Our research group has utilized a specific NAC protocol derived from the Allergic Rhinitis - Clinical Investigator Collaborative (AR-CIC) since 2015, and this protocol has been validated across a range of allergens [12–14, 41–43]. However, we have yet to publish a comprehensive examination of the pathophysiology associated with AR to ragweed pollen.

The objective of this study is to utilize our established protocol to elucidate the anticipated outcomes associated with the NAC following exposure to ragweed pollen extract. Through this investigation, we aim to provide a comprehensive understanding of what can be expected when employing our protocol in practice. The Allergic Rhinitis Microbiome (ARMS) study was designed to investigate and study ragweed–induced AR in humans using a NAC. The microbiome-related findings from ARMS will be published in a later manuscript.

## Methods

### Study design

**Fig. 1:**
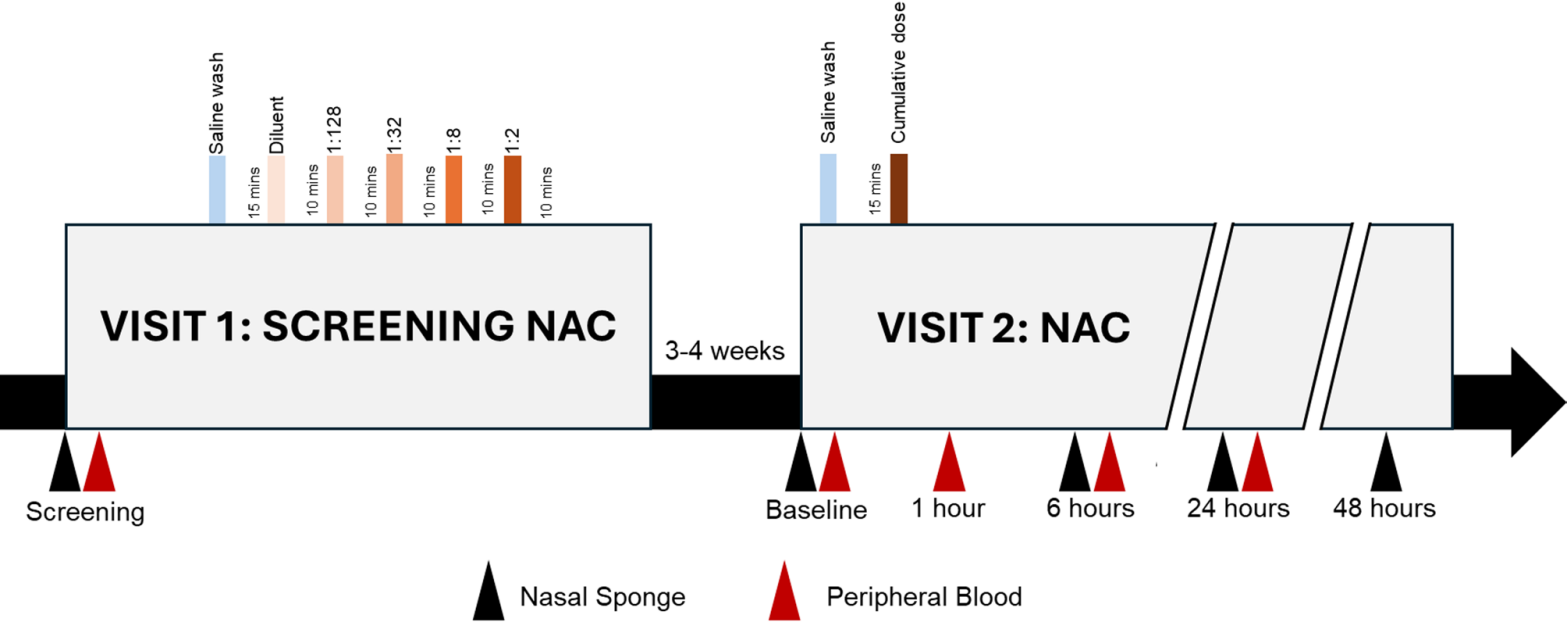
The Allergic Rhinitis Microbiome (ARMS) experimental design. The screening visit involves a titrated allergen challenge where the qualifying allergen dilution was determined using incremental concentrations of ragweed pollen allergen until ragweed pollen–sensitized participants reached the qualifying criteria (total nasal symptom score (TNSS) of ≥ 8/12 and peak nasal inspiratory flow (PNIF) fall of ≥ 50%). During the NAC visit, participants were challenged with a single cumulative allergen dilution equivalent to the sum of each dilution they received during screening. Nonallergics received the greatest allergen concentration (1:2). Various biological samples were collected throughout the study including at Screening and Baseline, and 1- hour, 6-hours, 24-hours, and 48-hours after allergen exposure

This study was approved by the Queen’s University Health Sciences and Affiliated Teaching Hospitals Research Ethics Board. All study participants provided informed written consent before participating in the study. The ARMS study was performed outside of the Southeastern Ontario ragweed pollen season and consisted of four visits: a screening visit, a cumulative single-dose NAC visit (Fig. 1) using ragweed pollen extract (Short Ragweed Pollen Allergen Extract, ALK Pharmaceuticals Inc, Ontario, Canada), and two follow-up visits. The stock solution concentration of ragweed pollen extract was 300.38 Allergen Extract (AgE) units/mL (1:20 weight/volume), consistent with our published protocols from the AR-CIC [12–14, 41, 42, 44] and in line with the European Academy of Allergy and Clinical Immunology (EAACI) Position paper [40] on the standardization of nasal allergen challenges and the 2023 AAAAI Rhinitis, Rhinosinusitis, and Ocular Allergy Committee Work Group Report [45]. As part of the screening visit, eligible participants underwent a titrated NAC. Each participant first received a wash of the nasal cavity with 5 mL of 0.9% sterile saline and then recorded their baseline allergy symptoms after 15 min, including total nasal symptom score (TNSS) and peak nasal Inspiratory Flow (PNIF) using an In-Check PNIF meter (Clement Clarke International Ltd, Harlow, United Kingdom). Next, a placebo challenge with 100 µL extract diluent (ALK Pharmaceuticals Inc, Ontario, Canada) was administered to both nostrils using the Pfeiffer Bidose Nasal Delivery Device (Aptar Pharma, Congers, New York). Symptoms and PNIF were recorded approximately 15 min after the diluent challenge. Participants sensitized to ragweed extract who did not have a hypersensitive response, defined here as a TNSS > 2 after receiving the placebo challenge, received a series of increasing concentrations of ragweed pollen extract: 1:128, 1:32, 1:8, and 1:2 (Table S1). Each allergen dilution was administered at a volume of 100 µL per nostril. TNSS and PNIF were measured 10 min after delivery of each allergen dilution. When the qualifying criteria (TNSS ≥ 8 and PNIF fall ≥ 50%) was achieved, the titrated challenge was stopped, and the last dilution of ragweed pollen extract received was classified as the qualifying concentration. If an allergic participant reached the highest concentration of 1:2 without qualifying, they were excluded. All nonallergic controls received a 1:2 dilution of ragweed pollen extract after the diluent challenge and required a TNSS ≤ 2 to proceed to the next visit.

Study participants who met the qualifying criteria were invited to return for the second visit 21 to 28 days after screening. Symptoms (and TNSS) and PNIF were recorded by the participants before the challenge. Next, in contrast to the screening visit, a single, personalized, cumulative allergen dilution (sum of all allergen dilutions received at screening) of ragweed pollen extract was delivered to both nostrils. Symptoms and PNIF were recorded incrementally at 15 min, 30 min, 1 h, and hourly until 12 h after the challenge. Symptoms and PNIF were collected again 24 h and 48 h after NAC.

### Study participants

Participants allergic to ragweed pollen allergen and nonallergic individuals (aged 18–65 years) were recruited from Kingston, Ontario, and the surrounding region. Participants were recruited using multiple methods, including searching the Kingston Allergy Research database for previous study participants, advertising, and posters displayed in allergy clinics. All medications taken within 30 days of screening and throughout the study were documented. Antihistamines, corticosteroids, and other anti-allergic medications were restricted for various time periods before each study visit (Table S2, Table S3).

### Inclusion and exclusion criteria

Allergic participants were recruited based on a self-reported clinical history of ragweed pollen-induced seasonal AR symptoms for at least two years. Sensitized participants, as defined by a positive ragweed pollen skin prick test (wheal diameter ≥ 3 mm above negative control of extract diluent), were included. Ragweed pollen allergic participants were also tested to a panel of locally common aeroallergens (Timothy grass, birch, alder, house dust mite mix (*Dermatophagoides pteronyssinus* and *Dermatophagoides farinae*), cat pelt, and dog hair) using the skin prick test. Nonallergic participants were required to have a negative skin prick test to all allergens on the same panel of aeroallergens, including ragweed pollen extract.

Participants were excluded from the ARMS study if they were actively trying to conceive, were pregnant, or lactating; had clinically significant nasal structural abnormalities or severe asthma requiring high-dose corticosteroids or a biologic therapy; were being treated with a ragweed allergen immunotherapy (AIT) or concluded a course of ragweed AIT in the last 3 years; had received an investigational product within the last 30 days; were a current tobacco smoker or had a history of smoking within the last 5 years; had a history of respiratory tract infection within the two weeks before any visit in this study. A TNSS greater than 2 before any allergen challenge (at screening and/or prior to the baseline NAC) was reason for exclusion since it indicated either AR from confounding allergies or non-specific hypersensitivity. Participants were also excluded if they had a history of human immunodeficiency virus, tuberculosis, hepatitis B or hepatitis C, or a history of clinically significant hematological, renal, endocrine, pulmonary, gastrointestinal, cardiovascular, hepatic, psychiatric or neurologic disease, or malignancies within the last 5 years that, in the opinion of the investigator, would interfere with the study evaluations or optimal participation in the study.

### Symptom diary cards

Participants received training on how to rate their symptoms accurately using symptom diary cards. Symptoms of runny nose/post-nasal drip, nasal congestion/stuffiness, sneezing, nasal itching, itchy/watery eyes, red/burning eyes, and itching of the ears/palate/throat were self-rated by each participant on 4-point scale in increasing severity (Table S4). Participants self-assessed their ocular symptoms, specifically redness with a handheld mirror. PNIF was also used to measure nasal airflow for each participant. The highest value of three PNIF measurements at a specific time was used.

### Blood collection and processing

Venous peripheral blood was collected from each participant at the screening visit and during the NAC visit at baseline, 1 h, 6 h, and at the 24-hour follow-up visit after NAC. Our internal laboratory at Kingston Health Sciences Centre - KGH Site conducted a complete blood count test with differential.

For serum analysis, blood was allowed to clot for a minimum of 30 min at room temperature and then centrifuged at 1500 g for 15 min before being aliquoted and frozen at -80 °C. Ragweed pollen sIgE and total IgE (tIgE) levels were measured with ImmunoCAP assays (Somagen Diagnostics Inc, Edmonton, Alberta, Canada) using a Phadia 100 system (Thermo Fisher Scientific, Waltham, Massachusetts).

### Nasal lavage collection and processing

Nasal lavage samples were collected from all participants during the NAC visit at baseline, 6 h, and 24 h after the beginning of the challenge and processed as previously described by Rawls et al. [12]. Cells from the nasal lavage were differentially stained using a Diff-Quick staining set (Siemens, Munich, Germany) and counted using a light microscope by at least 2 trained laboratory members. Neutrophils, eosinophils, macrophages, lymphocytes, basophils, and epithelial cells were counted and recorded until reaching a minimum of 250 cells per slide.

### Nasal sponge collection and processing

#### Nasal sponge collection

Nasal sponge samples were collected from participants at the screening visit; the NAC visit at baseline and 6 h after allergen exposure; and at 24 and 48 h post-NAC at their respective follow-up visits. Participants were instructed to sit with their heads against the wall. A sterile 3.5 cm x 0.6 cm x 1.2 cm sponge Merocel© Kenney Sinus-Pak (Medtronic, Dublin, Ireland) was placed along the middle meatus of one nostril for 5 min. Upon removal from the nostril, the sponge was placed in a chilled, sterile 15-mL Polypropylene elution tube containing 1 mL sterile saline. The tube was immediately placed on ice for one hour. Sponges were inserted into the empty barrel of a sterile 5 mL syringe inside a 15 mL Polypropylene tube and centrifuged at 1500 g for 15 min at 4 ˚C. The eluent was aliquoted and frozen at -80 ˚C until analysis.

#### Nasal cytokine processing

Cytokine levels were measured from one sponge aliquot using the Milliplex xMAP Human Cytokine/Chemokine Magnetic Bead Panel Multiplex Assay (MilliporeSigma from Merck KGaA, Darmstadt, Germany) on a Bio-Plex™ 200 system (Luminex^®^ xMAP multiplex technology, Bio-Rad Laboratories Ltd, Mississauga, Canada), according to the manufacturer’s protocol. The serum matrix was substituted for saline per the manufacturer’s recommendation, and thus the Standard and Quality Controls were reconstituted in saline. The following analytes were assessed: IL-1β, IL-4, IL-5, IL-6, IL-10, Il-13, IFN-γ, MCP-1, MIP-1β, RANTES, TNF-α,

### Statistical analysis

GraphPad Prism 10.4.0 was used to perform all statistical tests and generate all figures. Two-way repeated measures ANOVA with Šídák’s multiple comparisons test were used to evaluate the mean at each time point between allergic and nonallergic participants for TNSS and the percent PNIF fall from baseline. Friedman’s test with Dunn’s multiple comparison tests was used to assess differences within groups over time for all other samples. Mann-Whitney U tests were used to evaluate the significance of between-group comparisons for each sample. Correlations between two variables were studied using Spearman’s correlation. The threshold of significance was 0.05.

## Results

### Titration challenge NAC

Twenty-four ragweed-sensitized participants and 15 nonallergic controls completed a titrated NAC. It was confirmed that sensitized participants had significantly larger ragweed pollen wheals (*P* ≤ 0.0001, Figure S1A), higher peak TNSS (*P* ≤ 0.0001, Figure S1B), and a greater fall in PNIF (*P* ≤ 0.0001, Figure S1C) compared to controls. Twenty-two sensitized participants met the ARMS Screening NAC study criteria (TNSS ≥ 8, PNIF fall ≥ 50%), and all had positive skin prick test results for ragweed pollen. Two sensitized participants failed to meet the ARMS Screening NAC study criteria and were disqualified from the study. Three qualifying allergic participants and three nonallergic participants were lost to follow-up.

### ARMS participant demographics

Nineteen allergic and 12 nonallergic participants completed the cumulative single-dose NAC visit (Table S4). There were no significant differences in sex or age between the groups. The allergic group had a significantly larger wheal size (9.60 mm, SD = 5.51) compared to nonallergic controls (0.0 mm) (*P* ≤ 0.0001).

### Clinical symptoms induced by NAC in allergic participants

**Fig. 2:**
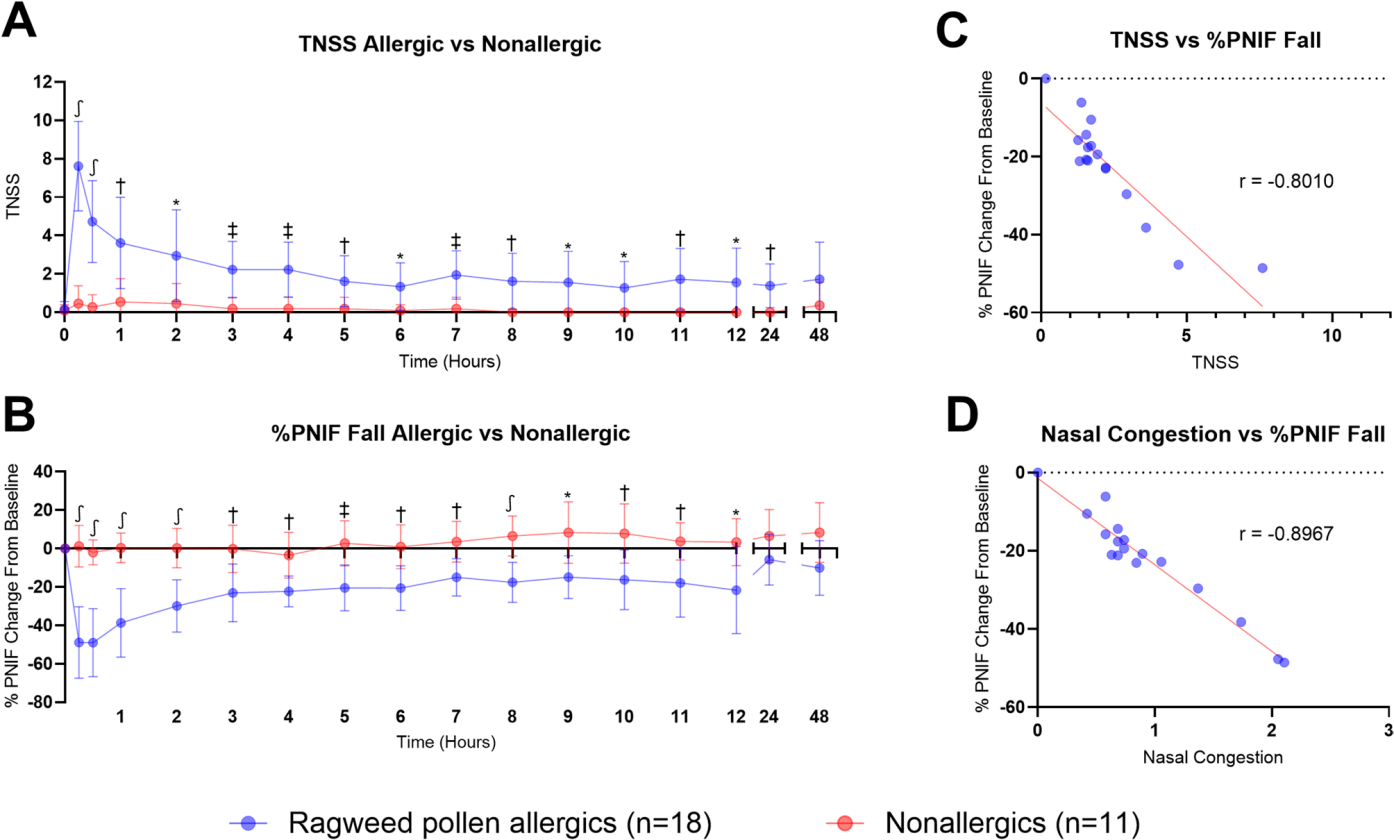
Clinical symptoms induced by nasal allergen challenge (NAC) with Ragweed pollen. (**A**) Mean total nasal symptom score (TNSS) was significantly greater at 15 min, 30 min, 1 h, 2 h, 3 h, 4 h, 5 h, 6 h, 7 h, 8 h, 9 h, 10 h, 11 h, 12 h, and 24 h after challenge when comparing allergic participants to nonallergic controls (*P* ≤ 0.05, two-way ANOVA with Šídák’s multiple comparisons test). (**B**) The mean PNIF fall from baseline was significantly greater at 15 min, 30 min, 1 h, 2 h, 4 h, 5 h, 6 h, 7 h, 8 h, 9 h, 10 h, 11 h, and 12 h for allergic participants after NAC than the nonallergic controls (*P* ≤ 0.05, two-way ANOVA with Šídák’s multiple comparisons test). (**C**) Allergic participants demonstrated a strong inverse correlation between TNSS and percent PNIF fall during the NAC (Spearman *r* = − 0.8010; *P* = 0.0002). (**D**) A strong inverse correlation was observed between these two clinical symptoms (Spearman *r* = − 0.8967; *P* ≤ 0.0001), suggesting that participants accurately reported their nasal symptoms. * = *P* ≤ 0.05; † = *P* ≤ 0.01; ‡ = *P* ≤ 0.001; ∫ = *P* ≤ 0.0001. *Error bars represent the mean and standard deviation*

The mean TNSS and the percent fall in PNIF from baseline were plotted for both allergic and nonallergic participants during the NAC visit (Fig. 2A and B). One allergic and one nonallergic participant was excluded from the analysis due to incomplete symptom diary entries.

The mean TNSS was significantly higher in participants with ragweed pollen allergy compared to nonallergic controls at multiple time points following the NAC (Fig. 2A). Specifically, TNSS was significantly greater at 15 min (*P* ≤ 0.0001), 30 min (*P* ≤ 0.0001), 1 h (*P* = 0.0003), 2 h (*P* = 0.0063), 3 h (*P* = 0.0004), 4 h (*P* = 0.0003), 5 h (*P* = 0.0097), 6 h (*P* = 0.0100), 7 h (*P* = 0.0005), 8 h (*P* = 0.0037), 9 h (*P* = 0.0131), 10 h (*P* = 0.0165), 11 h (*P* = 0.0047), 12 h (*P* = 0.0306), and 24 h (*P* = 0.0014) after the NAC.

Similarly, the mean percent PNIF fall from baseline were also plotted during the cumulative single-dose NAC visit (Fig. 2B). The mean percent PNIF fall from baseline was significantly greater in allergic participants at several time points, including 15 min (*P* ≤ 0.0001), 30 min (*P* < 0.0001), 1 h (*P* ≤ 0.0001), 2 h (*P* ≤ 0.0001), 3 h (*P* = 0.0025), 4 h (*P* = 0.0051), 5 h (*P* = 0.0007), 6 h (*P* = 0.0012), 7 h (*P* = 0.0023), 8 h (*P* ≤ 0.0001), 9 h (*P* = 0.0113), 10 h (*P* = 0.0089), 11 h (*P* = 0.0043), and 12 h (*P* = 0.0115) compared to nonallergic controls.

Significant inverse correlations were observed between both TNSS (Spearman *r* = -0.8010, *P* = 0.0002) and nasal congestion (Spearman *r* = -0.8967, *P* ≤ 0.0001) with percent PNIF fall from baseline among allergic participants (Fig. 2C, D).

Likewise, TRSS (Figure S2) was significantly elevated after NAC when comparing ragweed pollen allergic to nonallergic participants at 15 min (*P* < 0.0001), 30 min (*P* < 0.0001), 1 h (*P* = 0.0008), 2 h (*P* = 0.0083) 3 h (*P* = 0.0136), 4 h (*P* = 0.0256), 8 h (*P* = 0.0125), 9 h (*P* = 0.0244), 11 h (*P* = 0.0125), 12 h (*P* = 0.0346), and 24 h (*P* = 0.0031).

In addition, the mean TOSS (Figure S2) was significantly greater in participants with ragweed pollen allergy than in the control group at 15 min (*P* = 0.0015) after the allergen challenge.

### Nasal inflammatory cellular influx and egress following the NAC

**Fig. 3:**
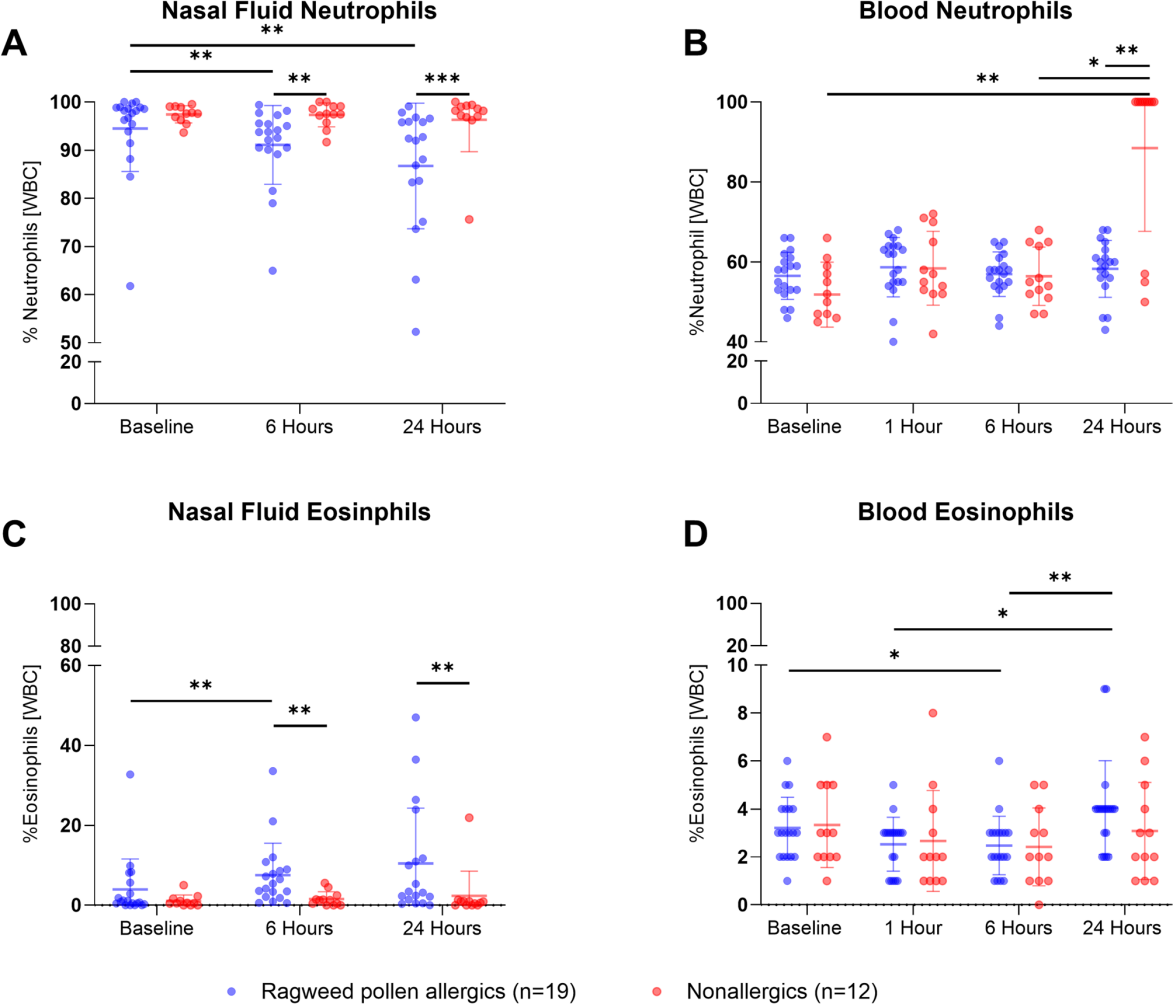
Influx and regress of white blood cells (WBC) in the nose and peripheral blood following nasal allergen challenge (NAC) with Ragweed pollen. Mean cell counts of nasal and blood neutrophils (**A, B**) and eosinophils (**C, D**) are shown as percentages (%) of WBC. Allergic participants had significantly lower nasal neutrophil levels at 6 and 24 h after NAC compared to nonallergic participants (*P* ≤ 0.05, Mann-Whitney U test). Nasal neutrophil levels also decreased at 6 and 24 h in allergic participants compared to baseline (*P* ≤ 0.05, Friedman test with Dunn’s post-test). Nasal eosinophils were higher in allergic participants at 6 and 24 h after allergen exposure (*P* ≤ 0.05, Mann-Whitney U test) and significantly increased at 6 h compared to baseline (*P* ≤ 0.05, Friedman test with Dunn’s post-test). Blood neutrophil counts were elevated in nonallergic participants at 24 h post-challenge and significantly higher at 24 h compared to baseline and 6 h (*P* ≤ 0.05 Mann-Whitney U test). Blood eosinophil counts were similar between allergic and nonallergic participants (*P* ≥ 0.05) but increased at 24 h after NAC in allergic participants (*P* ≤ 0.05, Friedman test with Dunn’s post-test). A significant decrease in blood eosinophils was observed at 6 h compared to baseline in allergic participants (*P* ≤ 0.05, Friedman test with Dunn’s post-test). *P ≤ 0.05; **P ≤ 0.01; ***P ≤ 0.001; ****P ≤ 0.0001. Error bars represent the mean and standard deviation

White blood cell counts in nasal fluid and peripheral blood were assessed at baseline, 6 h, and 24 h post-NAC (Fig. 3). Additionally, peripheral blood was collected at 1-hour post-NAC.

In nasal fluid, neutrophils were the predominant cell type. Following allergen exposure, allergic participants’ neutrophils (as a percentage of white blood cells, Fig. 3A) decreased from baseline (94.49%) to 6 h (91.10%) and 24 h (87.97%), while eosinophil percentages increased (baseline: 3.95%, 6 h: 7.51%, 24 h: 9.50%, Fig. 3B). Nonallergic participants had significantly higher neutrophil counts at 6 h (*P* = 0.0019) and 24 h (*P* = 0.0004). Allergic participants had significantly higher eosinophils at 6 h (*P* = 0.0010) and 24 h (*P* = 0.0049). Within allergic participants, neutrophil levels significantly decreased (*P* = 0.0081) and eosinophil levels significantly increased (*P* = 0.0061) at 6 h compared to baseline. Nasal eosinophil counts were weakly correlated with TNSS scores (Spearman *r* = 0.3668, *P* = 0.0069, Figure S3). There was no significant difference in the levels of monocytes or lymphocytes between allergic and nonallergic participants (*P* ≥ 0.05, data not shown). No basophils were found in the nasal fluid of allergic or nonallergic participants (data not shown).

In peripheral blood, nonallergic participants had significantly higher neutrophil percentages at 24 h compared to baseline (*P* = 0.0012) and 6 h (*P* = 0.0160) (Fig. 3C). No significant differences were observed in eosinophil percentages between the groups (*P* ≥ 0.05, Fig. 3D). In allergic participants, eosinophil percentages decreased significantly at 6 h compared to baseline (*P* = 0.0499) and at 6 h compared to 24 h (*P* = 0.0065).

### Total IgE and ragweed pollen sIgE during the NAC

**Fig. 4:**
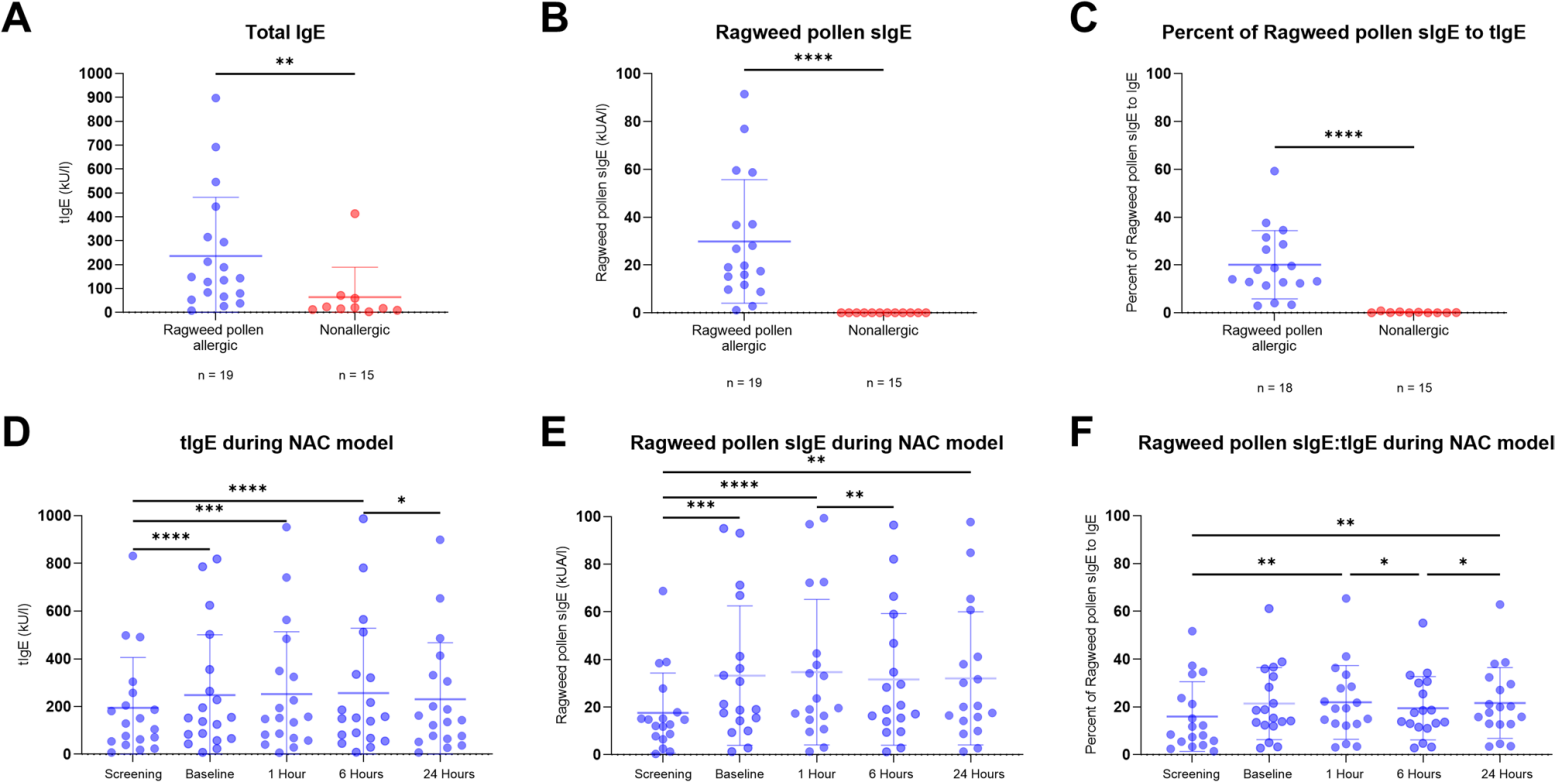
Temporal changes in total IgE, ragweed-specific IgE, and the tIgE/sIgE ratio following nasal allergen challenge (NAC). The mean total IgE (tIgE) and ragweed-specific IgE (sIgE) concentrations, along with the tIgE/sIgE ratio, were significantly higher in allergic participants compared to nonallergic controls (*P* ≤ 0.05, Mann-Whitney U test) (**A-C**). One allergic participant was excluded from time-dependent changes in tIgE and sIgE concentrations were further analyzed in allergic participants across five time points during the (**D-F**). tIgE was significantly elevated at baseline, 1 h, and 6 h after NAC compared to screening samples (*P* ≤ 0.05, Friedman test with Dunn’s post-hoc test), with a significant decrease from 6 to 24 h post-NAC (*P* ≤ 0.05). sIgE was significantly increased at baseline, 1 h, and 24 h post-NAC compared to screening (*P* ≤ 0.05), and significantly decreased between 1 and 6 h post-NAC (*P* ≤ 0.05). The tIgE/sIgE ratio was significantly higher at 1 h and 24 h post-NAC compared to screening (*P* ≤ 0.05), with a significant decrease at 6 h (*P* < 0.05). * = *P*  ≤ 0.05; ** = *P* ≤ 0.01; *** = *P* ≤ 0.001; **** = *P* ≤ 0.0001. Error bars represent the mean and standard deviation

Serum tIgE was measured at screening and at the cumulative single-dose NAC (baseline, 1 h, 6 h, and 24 h after NAC). Allergic participants had a mean tIgE concentration of 236.4 kU/L (*n* = 19, SD = 245.1), significantly higher (*P* < 0.01) than 64.17 kU/L measured in nonallergic participants (*n* = 12, SD = 124.6) (Fig. 4A). Similarly, allergic participants had significantly higher ragweed-sIgE (29.80 kUA/L, *n* = 18, SD = 25.84) than nonallergic participants (0.01 kUA/L, SD = 0.01, *P* ≤ 0.0001) (Fig. 4B). The sIgE/tIgE ratio was significantly higher in allergic participants (20.1%, *n* = 18, SD = 14.3) compared to nonallergic participants (0.18%, SD = 0.26) (Fig. 4C).

At all time points, allergic participants showed an increase in mean tIgE concentrations: 194.3 kU/L (screening), 248.0 kU/L (baseline NAC), 252.3 kU/L (1 h), 256.9 kU/L (6 h), and 230.6 kU/L (24 h) (Fig. 4D). Significant increases were observed at baseline, 1 h, and 6 h compared to screening (*P* ≤ 0.0001), with a decrease between 6 and 24 h (*P* = 0.0208).

For ragweed-sIgE, allergic participants had mean concentrations of 17.5 kUA/L (screening), 33.18 kUA/L (Baseline), 34.63 kUA/L (1 h), 31.61 kUA/L (6 h), and 32.05 kUA/L (24 h) (Fig. 4E). Significant increases were observed at baseline, 1 h, and 24 h (*P* ≤ 0.0001), with a decrease from 1 h to 6 h (*P* = 0.0034).

The sIgE/tIgE ratio for allergic participants was 15.98% (screening), 21.39% (baseline NAC), 21.89% (1 h), 19.47% (6 h), and 21.60% (24 h) (Fig. 4F). Significant increases were seen at 1 h and 24 h (*P* = 0.0022, *P* = 0.0034), with a decrease at 6 h compared to both 1 h and 24 h (*P* = 0.0224, *P* = 0.0316).

### Nasal cytokine profile following the NAC

**Fig. 5:**
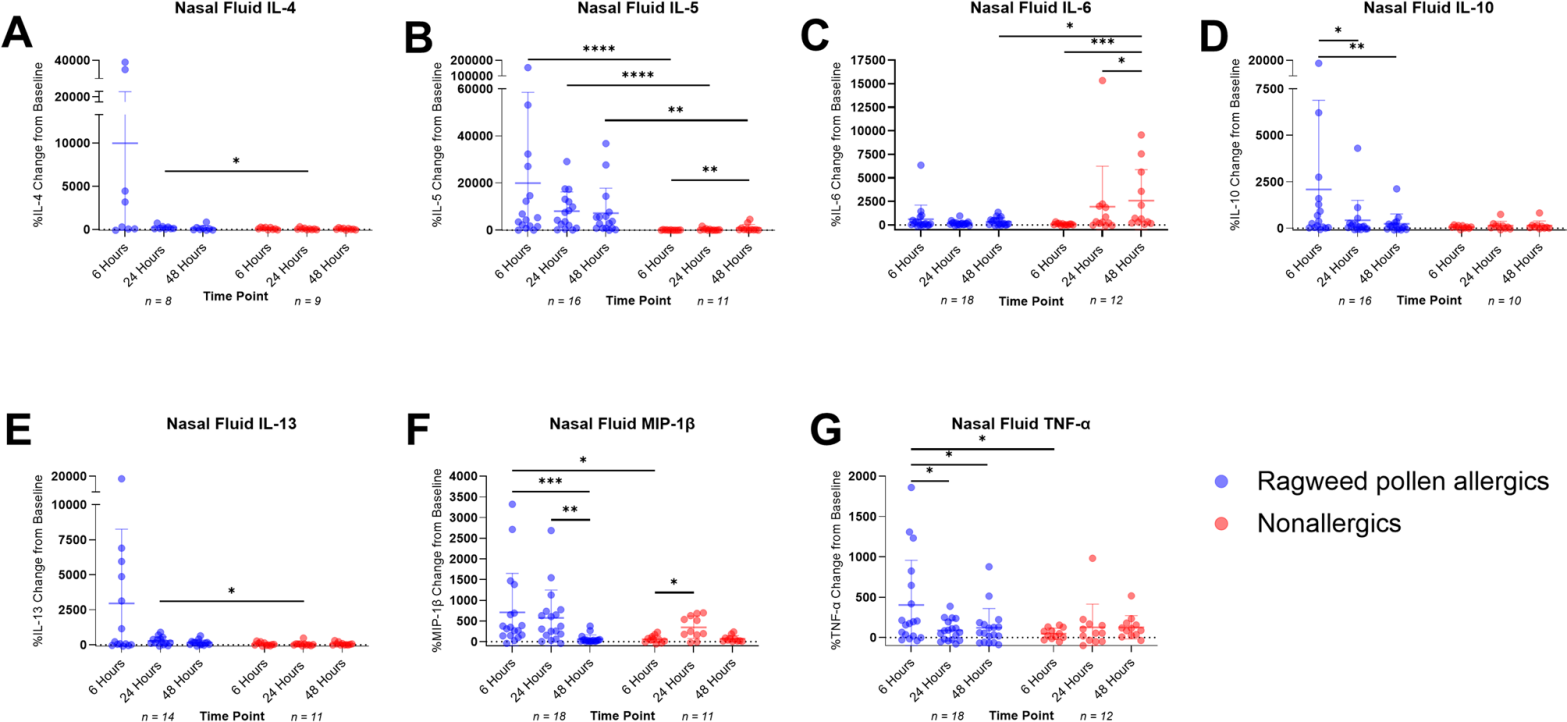
Cytokine profiles following nasal allergen challenge (NAC). The cytokine concentrations of various cytokines IL-4 (**A**), IL-5 (**B**), IL-6 (**C**), IL-10 (**D**), IL-13 (**E**), MIP-1β (**F**) and TNF-α (**G**) were plotted as a percentage normalized to baseline for each participant. IL-5, MIP-1β, and TNF-α were significantly higher in nasal fluid samples collected after challenge from allergic participants when compared to nonallergic controls at 6 h (*P* ≤ 0.05; Mann-Whitney U tests). The percent changes of IL-4, IL-5, and IL-13 were significantly greater in allergic participants compared to nonallergic controls at 24 h (*P* ≤ 0.05, respectively; Mann-Whitney U tests). Levels of IL-5 were still significantly elevated compared nonallergic controls at 48 h (*P* ≤ 0.05; Mann-Whitney U tests). Conversely, levels of IL-6 were significantly lower in allergic participants compared to healthy controls at 48 h post-NAC (*P* ≤ 0.05; Mann-Whitney U tests). Paired analyses demonstrated that the percent change of TNF-α and IL-10 from baseline was significantly elevated at 6 h compared to 24 h and 48 h (*P* ≤ 0.05, Friedman test with Dunn’s multiple comparison test) post-challenge among allergic participants. Likewise, there were significant decreases in normalized MIP-1β concentrations at 48 h compared to other timepoints (*P* ≤ 0.05; Friedman test with Dunn’s multiple comparison test). The percent change IL-5 and IL-6 was elevated at 48 h post-NAC compared to 6 h and 24 h (*P* ≤ 0.05, Friedman test with Dunn’s multiple comparison test) among nonallergic participants. * *P* ≤ 0.05; ** = *P* ≤ 0.01; *** = *P* ≤ 0.001; **** = *P* ≤ 0.0001. Error bars represent the mean and standard deviation

Cytokine responses were significantly different between allergic and nonallergic participants post NAC (Fig. 5). In general, allergic participants showed a marked increase in cytokine levels (normalized to baseline) after the NAC, while nonallergic participants showed minimal changes. There was no significant difference in IFN-γ, MCP-1, and RANTES levels between allergic and nonallergic participants (*P* ≥ 0.05) at any timepoint post-NAC (data not shown). At 6 h, allergic participants had significantly higher IL-5 (*P* ≤ 0.0001), MIP-1β (*P* = 0.0006), and TNF-α (*P* = 0.0346) compared to nonallergic participants. At 24 h, IL-4 (*P* = 0.0254), IL-5 (*P* ≤ 0.0001), and IL-13 (*P* = 0.0211) remained elevated, with IL-5 significantly higher at 48 h (*P* = 0.0068). IL-6 was significantly lower at 48 h in allergic participants compared to nonallergic participants (*P* = 0.0193).

Among allergic participants, IL-10 and TNF-α levels were significantly higher at 6 h than at 24 h (*P* = 0.04, *P* = 0.0373) and 48 h (*P* = 0.0023, *P* = 0.0373). MIP-1β levels were higher at 6 h (*P* = 0.0002) and 24 h (*P* = 0.0014) compared to 48 h. Nasal eosinophil counts were weakly correlated with IL-5 concentrations (Spearman *r* = 0.3225, *P* = 0.0063, Figure S4).

Nonallergic participants showed increased IL-6 (*P* = 0.0429) and MIP-1β (*P* = 0.0315) at 24 h compared to 6 h. IL-5 (*P* = 0.0060) and IL-6 (*P* = 0.0007) were elevated at 48 h compared to 6 h. No other cytokine differences were observed (*P* ≥ 0.05).

## Discussion

This study successfully employed the AR-CIC’s NAC protocol to induce AR symptoms in ragweed pollen-allergic participants. Allergic individuals experienced significantly higher TNSS scores and greater reductions in PNIF compared to nonallergic participants. As demonstrated in previous studies, allergic participants had an immediate increase in TNSS, followed by gradual symptom relief, while PNIF remained lower throughout [12–14, 43, 46]. Still, TNSS was significantly elevated compared to nonallergic participants until 24 h after the NAC. A strong inverse correlation between TNSS and PNIF fall (Spearman *r* = -0.8010; *P* = 0.0002) confirmed that allergic participants accurately reported their symptoms. At 48 h post-exposure, TNSS remained elevated, representing the first demonstration of NAC-induced AR symptoms at this time point. While allergic participants did not experience significantly different symptoms compared to nonallergic participants at 48 h, biological changes appeared to continue. This is supported by the observed increase in specific cytokines, IL-4, IL-5, IL-10, IL-13, MIP-1β, and TNF-α, in the nasal mucosa. However, it is important to note that extending the observation period increases the likelihood of participants being exposed to perennial allergens, which could confound the results for polysensitized participants. Overall, these findings support the AR-CIC protocol as a reliable tool for studying AR and raise important questions about the duration of symptoms following a nasal allergen challenge.

The NAC has emerged as a powerful translational research tool in AR. Despite its utility, NACs are not yet standardized internationally nor formally recognized by regulatory agencies like the FDA. Nevertheless, a growing consensus on best practices is emerging. For example, the 2023 AAAAI Rhinitis, Rhinosinusitis, and Ocular Allergy Committee published a Work Group Report outlining practical NAC approaches across the US and Europe [45], while EAACI has also issued position papers emphasizing the need for standardized protocols in clinical practice [40]. Although these documents primarily target clinical application, their principles are widely adopted in research contexts. The lack of unified standards—especially around allergen dose, dilution, application technique, and objective outcome measurement—has limited cross-study comparability.

To address these gaps, Canadian initiatives such as the AR-CIC have developed optimized NAC protocols [12–14, 41, 42, 44]. These efforts are crucial for generating reproducible, high-quality data on the pathophysiology of AR and for evaluating new treatments. In our study, the use of a titrated NAC protocol—employing serial dilutions of ragweed pollen extract—was designed to reflect both safety considerations and clinical relevance [40, 45]. By providing a detailed description of our protocol, we aim to promote replicability and encourage the adoption of NACs in both clinical and research settings.

Given their ability to model both the early- and late-phase allergic responses in a controlled manner [16, 20, 22, 27, 28, 31, 34, 47], NACs represent a valuable diagnostic and investigational tool [45]. From a research standpoint, NACs offer a unique window into immune activation, mediator release, and cellular recruitment, providing a dynamic and robust model of allergic inflammation [48]. With increasing interest in precision medicine and phenotyping in AR [5], standardizing and promoting NAC use across disciplines will enhance our ability to translate mechanistic insights into meaningful clinical interventions.

Eosinophil dynamics consistent with the AR response can be seen in this study. Serum eosinophil levels decreased at 1- and 6-hours post-NAC, followed by a rebound at 24 h. Conversely, eosinophil counts in nasal fluid increased at 6 h and remained elevated at 24 h. This inverse relationship of decreased eosinophils in the blood and increased eosinophils in the nasal fluid supports the idea that blood eosinophils become activated and extravasate into the nasal tissues during allergen exposure. Although this phenomenon has been documented in previous NAC studies [49, 50], direct evidence of eosinophil migration remains scarce [51, 52]. Our study did not assess eosinophil activation or degranulation, such as ECP release, which could have provided more insight into the functional role of eosinophils in AR. Since it is known that different eosinophilic conditions have a marked heterogeneity in degranulation [53, 54], future studies should address these gaps to better understand the mechanisms driving severe disease phenotypes.

Regarding sIgE, AR participants had significantly higher levels than nonallergic controls. While participants were not required to meet a specific sIgE threshold for inclusion, the mean sIgE in ARMS across all timepoints was 29.80 kUA/L, suggesting high sensitization to ragweed according to the IgE classes, whereby class 4 or grade 4 is equivalent to 17.5-52.5kUA/L. Notably, the levels of sIgE significantly increased after the screening NAC and remained elevated at baseline-NAC, with a slight decrease at 6 h post-exposure. Previous studies have demonstrated that elevated sIgE levels persist for over a month after allergen exposure [12, 33, 53, 54]. These findings are particularly relevant for future studies using the NAC to examine sIgE or for developing novel therapies targeting sIgE. Additionally, these findings suggest that future biological sampling before the cumulative single-dose NAC is necessary to understand better the long-term effects of allergen exposure and the presence of subclinical inflammation without observable symptoms or minimal persistent inflammation [55]. 

Cytokine expression following NAC demonstrated notable upregulation of both Th1- and Th2 cytokines in allergic participants, with elevated levels of IL-4, IL-5, IL-10, IL-13, MIP-1β, and TNF-α, compared to nonallergic participants. The observed lack of consistent change in several cytokines—including IFN-γ, MCP-1, and RANTES—across time points and between allergic and nonallergic participants warrants further consideration. One limitation of our study is the absence of a time point prior to the 6-hour mark post-NAC, which may have precluded the detection of early-phase cytokine responses, many of which are known to peak within 1–3 h following allergen exposure [6–11, 56]. Moreover, individual variability in the timing and intensity of cytokine responses may have further diluted group-level effects. Previous studies suggest that only ~ 50% of allergic individuals exhibit a measurable late-phase allergic response, which supports the heterogeneity of cytokine expression across NAC studies [27, 57–59]. 

Among allergic participants, IL-5 expression was significantly higher than in nonallergic participants at all time points post-challenge, which aligns with the known role of IL-5 in AR pathophysiology and previous NAC studies. IL-5 promotes eosinophil maturation, survival, and activation and has been shown to peak at 6 h in previous NAC studies with ragweed and other allergens [15, 38]. Previous studies have demonstrated a strong positive correlation between IL-5 concentrations and nasal eosinophil counts [23]. We did not find a strong correlation between IL-5 levels and nasal eosinophil counts supporting the role of IL-5 on eosinophil biology. This weak correlation may be due to the small sample size, warranting further investigation in larger studies.

Nasal IL-4 levels were low in both allergic and nonallergic participants, with no significant differences between the two groups. This aligns with findings from previous NAC studies that reported similarly low IL-4 levels in nasal samples [6, 15, 38]. While some studies have observed increases in IL-4 during later stages of the NAC [16, 60], our results suggest that our temporal sampling may not have fully captured the IL-4 response. The low levels of IL-4 in our study may also be explained by IL-4 binding to its receptor on nasal cells [61, 62] or by the presence of splice variants such as IL-4 δ2, which may affect the overall response [63]. Additionally, IL-4 may migrate to the bloodstream, where it interacts with circulating B cells. Thus, further research should explore serum IL-4 expression, and the role of IL-4 splice variants in AR.

## Conclusions

In summary, this study confirmed the utility of the AR-CIC NAC protocol in inducing clinical and immune responses in ragweed pollen-allergic participants. The findings contribute to our understanding of eosinophil dynamics, IgE production, and cytokine expression in AR, but further studies are needed to explore the functional capacity of eosinophils, the relationship between sIgE and clinical symptoms and the mechanisms underlying cytokine responses in AR. Future NAC studies should consider a more comprehensive time series of sIgE and IL-4 dynamics, as well as the activated eosinophils in the nasal mucosa, to provide a deeper understanding of allergic inflammation. This study population will serve as the basis for a future analysis investigating the impact of a NAC on the nasal microbiome.

## Electronic supplementary material

Below is the link to the electronic supplementary material.

Supplementary Material 1

## Data Availability

No datasets were generated or analysed during the current study.

## Abbreviations

AgE: Allergenic extract
AIT: Allergen immunotherapy
AR: Allergic rhinitis
AR-CIC: Allergic rhinitis – clinical investigator collaborative
ARMS: Allergic rhinitis microbiome study
IgE: Immunoglobulin–E
ILC2: Group 2 innate lymphoid cells
NAC: Nasal allergen challenge
PNIF: Peak nasal inspiratory flow
sIgE: Allergen specific immunoglobulin–E
TNSS: Total nasal symptom score
tIgE: Total immunoglobulin–E
WBC: White blood cells
