# Supplementary material for: Characterizing the symptomatology and pathophysiology of allergic rhinitis using a nasal allergen challenge model – a subset of the allergic rhinitis microbiome study
Source: Allergy Asthma Clin Immunol. 2025 Aug 18;21:36. doi: 10.1186/s13223-025-00980-5 (PMC12362900; doi:10.1186/s13223-025-00980-5)
Supplement: Supplementary file 1 — Supplementary Material 1 [file 13223_2025_980_MOESM1_ESM.docx]

Table S1. Allergen concentrations in the ARMS titration challenge.

| Dilution | Concentration*  [AgE/mL] | Total administered  [AgE/mL] |
| --- | --- | --- |
| 1:128 | 2.3 | 0.5 |
| 1:32 | 9.4 | 1.9 |
| 1:8 | 37.5 | 7.5 |
| 1:2 | 150.2 | 30.0 |

*Short Ragweed Pollen Allergen Extract, 300.38 AgE/mL, 1:20 weight/volume.

Table S2. Medications not permitted before or during the ARMS study.

| Medication | Washout Period |
| --- | --- |
| Beta-blockers | 14 days |
| Tricyclic Antidepressants | 14 days |
| Monoamine Oxidase Inhibitors | 14 days |
| Omalizumab, Mepolizumab, Reslizumab, Dupilumab or Benralizumab | 6 months |

Table S3. Restricted medications and washout periods before and during the ARMS study.

| Medication | Washout prior to visit 1 | Washout prior to visit 2,3,4 |
| --- | --- | --- |
| Systemic (intramuscular and/or intravenous and/or oral) corticosteroids (topical hydrocortisone ≤1% covering ≤ 10 % of body surface without occlusion is allowed) | 30 days | Prohibited for duration of study |
| Oral or parenteral antibiotics | 30 days | Prohibited for duration of study |
| Inhaled or intranasal corticosteroids | 21 days | Prohibited for duration of study |
| Topical Corticosteroids (Topical hydrocortisone ≤1 % covering ≤ 10 % of body surface without occlusion is allowed) | 7 days | 7 days |
| Anti-allergic ophthalmic treatments e.g. Patanol^®^, or Zaditen^®^ | 3 days | 3 days |
| Oral decongestants | 3 days | 3 days |
| H_1_-receptor antagonists (oral and/or topical) | 3 days | 5 days |
| Intranasal Decongestants | 24 hours | Prohibited for duration of study |
| Ocular drops (saline solution for contact lens use is allowed)* | 24 hours | 24 hours |

***** Participants may use these medications provided that the washouts are observed

| **Table S**4**. Symptom Diary Cards****Symptom Category** | **Symptom** | **Score 0 (None)** | **Score 1 (Mild)** | **Score 2 (Moderate)** | **Score 3 (Severe)** |
| --- | --- | --- | --- | --- | --- |
| TRSS (Total Rhinoconjunctivitis Symptom Score) | Itchy ears / palate / throat | Symptom absent | Symptom present but not bothersome | Symptom is bothersome but tolerable | Symptom is hard to tolerate, desiring treatment |
|  | Runny nose / post-nasal drip |  |  |  |  |
|  | Nasal congestion / stuffiness |  |  |  |  |
|  | Sneezing |  |  |  |  |
|  | Nasal itching |  |  |  |  |
|  | Itchy / watery eyes |  |  |  |  |
|  | Red / burning eyes |  |  |  |  |
| TNSS (Total Nasal Symptom Score) | Runny nose / post-nasal drip | As above | As above | As above | As above |
|  | Nasal congestion / stuffiness |  |  |  |  |
|  | Sneezing |  |  |  |  |
|  | Nasal itching |  |  |  |  |
| TOSS (Total Ocular Symptom Score) | Itchy / watery eyes | As above | As above | As above | As above |
|  | Red / burning eyes |  |  |  |  |

Table S4 – ARMS Study Demographics

| Demographic Parameter |  | Allergic Group | Nonallergic Group | p-value |
| --- | --- | --- | --- | --- |
| Number of Participants |  | 19 | 12 |  |
| Mean Age, years (SD) |  | 45.42 (11.27) | 42.00 (15.80) | 0.6674 |
| Biological sex at Birth, n (%) | Female | 11 (58) | 8 (67) | 0.7172 |
|  | Male | 8 (42) | 4 (33) |  |
| Race, n (%) | White | 17 (89.47) | 10 (83.33) |  |
|  | White and Indian | 0 | 1 (8.33) |  |
|  | White and American Indian or Alaska Native | 1 (5.26) | 0 |  |
|  | Chinese | 1 (5.26) | 1 (8.33) |  |
| Ragweed mean wheal size, mm (SD) |  | 13.82 (5.99) | 0.08 (0.28) | <0.0001 |
| Qualifying NAC dose, n (%) | 1:128 | 7 (36.84) |  |  |
|  | 1:32 | 7 (36.84) |  |  |
|  | 1:8 | 5 (26.32) |  |  |
|  | 1:2 | 0 |  |  |

Abbreviations: n, number; SD, Standard Deviation.

**Figure S1. Symptoms during the titrated allergen challenge**. The Ragweed pollen wheal size data (A) for sensitized (n = 24) and without allergy (n = 15) participants were collected during the screening visit. Mean wheal size was significantly greater in Ragweed pollen-sensitized participants than nonallergic controls (P ≤ 0.0001, Mann-Whitney U test). Ragweed pollen-sensitized participants also had significantly elevated peak total nasal symptom scores (TNSS) than nonallergic participants (P ≤ 0.0001, Mann-Whitney U test) (B). Likewise, the percent peak nasal inspiratory flow (PNIF) fall was significantly greater in Ragweed pollen-sensitized participants than in nonallergic controls (P ≤ 0.0001, Mann-Whitney U test) (C).

**Figure S2. Rhinoconjunctivitis symptoms induced by the nasal allergen challenge (NAC) with Ragweed pollen.** Mean total rhinoconjunctivital symptom scores (TRSS) (left), and total ocular symptom score (TOSS) (right) were plotted for the participants with and without allergy. Mean TRSS was significantly higher for allergic participants at 15 minutes (P < .0001), 30 minutes (P < .0001), 1 hour (P =.0008), 2 hours (P = .0083), 3 hours (P = .0136), 4 hours (P = .0256), 8 hours (P = .0125), 9 hours (P = .0244), 11 hours (P = .0125), 12 hours (P = .0346), and 24 hours (P = .0031) after challenge than nonallergic. For participants with Ragweed pollen allergy, the mean TOSS was significantly greater only at 15 minutes (P = 0.0015) after allergen challenge than the healthy controls (two-way ANOVA with Šídák's multiple comparisons test). * = P ≤ 0.05; † = P ≤ 0.01; ‡ = P ≤ 0.001; ∫ = P ≤ 0.0001.

**Figure S3. Nasal symptoms correlate with nasal eosinophil levels after the nasal allergen challenge (NAC).** Total Nasal Symptom Scores (TNSS) scores of ragweed allergic participants taken at the baseline, 6 hours post-NAC, and 24 hours post-NAC are plotted against the nasal eosinophils counts as a percentage of white blood cells (WBC) at the corresponding time points. Among allergic participants, a significant, weak correlation exists between TNSS and eosinophil counts in the nose (Spearman r = 0.3668, P = 0.0069).

**Figure S4. IL-5 and eosinophil levels are weakly correlated after the nasal allergen challenge (NAC).** The percent change in the concentration of IL-5 from the nasal fluid of ragweed allergic participants at 6 hours post-NAC and 24 hours post-NAC is plotted against the nasal eosinophils counts as a percentage of WBC at the corresponding time points. A weak correlation exists between nasal IL-5 concentrations and eosinophil counts (Spearman r = .3225, P = .0663).
